# Japanese Encephalitis Virus Genotype Replacement, Taiwan, 2009–2010

**DOI:** 10.3201/eid1712.110914

**Published:** 2011-12

**Authors:** Yi-Ying Chen, Yi-Chin Fan, Wu-Chun Tu, Rey-Yi Chang, Chen-Chang Shih, In-Houng Lu, Maw-Shien Chien, Wei-Cheng Lee, Ter-Hsin Chen, Gwong-Jen Chang, Shyan-Song Chiou

**Affiliations:** National Chung Hsing University, Taichung, Taiwan (Y.-Y. Chen, Y.-C. Fan, W.-C. Tu, M.-S. Chien, W.-C. Lee, T.-H. Chen, S.-S. Chiou);; National Dong Hwa University, Hualien, Taiwan (R.-Y. Chang, I.-H. Lu);; Mennonite Christian Hospital, Hualien (C.-C. Shih);; Centers for Disease Control and Prevention, Fort Collins, Colorado, USA (G.-J. Chang)

**Keywords:** Japanese encephalitis, genotype, pig farm, viruses, vector-borne infections, Taiwan

## Abstract

Genotype I of Japanese encephalitis virus first appeared in Taiwan in 2008. Phylogenetic analysis of 37 viruses from pig farms in 2009–2010 classified these viruses into 2 unique subclusters of genotype I viruses and suggested multiple introductions and swift replacement of genotype III by genotype I virus in Taiwan.

Japanese encephalitis virus (JEV), a mosquito-borne flavivirus, is a common cause of viral encephalitis in southern and eastern Asia. Pigs are a readily available virus amplifying host, and *Culex tritaeniorhynchus* mosquitoes, the primary transmission vector of JEV, breed predominantly in rice paddies (*1*). Molecular epidemiologic studies have found that the introduction of JEV into subtropical regions from tropical regions, possibly associated with the Pacific flyway of spring migratory birds, may contribute to annual JEV epidemics and epizootics in subtropical regions (*2*).

Phylogenetic reconstruction, based on capsid and precursor membrane or envelope (E) structural protein genes of JEV, supports an Indonesian origin and further classifies JEV into 6 genotypes (*3*,*4*). The genotype III (GIII) virus is most widely distributed in the temperate zone and is the genotype most frequently associated with JEV outbreaks and epidemics in eastern and Southeast Asian countries (*4*). Genotype I (GI) JEV, which originated in Indonesia and circulated in Thailand and Cambodia during the 1970s, appeared in South Korea and Japan during the 1990s. The replacement of GIII by GI was swift, completed in just a few years in Japan, South Korea, Thailand, and Vietnam (*2*,*5*–*7*). The introduction of GI JEV into Japan may have followed a south-to-north or west-to-east route and has been reported to have come from Southeast Asia and mainland People’s Republic of China (*8*). However, events leading to replacement, including introduction, transition dynamics, and interplay between the 2 virus genotypes, are still unclear. The only 2 remaining GI-free island countries, the Philippines and Taiwan, provide a location where the transmission dynamics of these 2 JEV genotypes can be elucidated. GI JEV was first detected in northern Taiwan during the winter of 2008 by the Centers for Disease Control, Taiwan (CDC-Taiwan) (*9*); however, GIII JEV remained the dominant virus in Taiwan that year.

## The Study

To understand early events leading to the replacement of GIII viruses by GI viruses in JEV-endemic areas, we conducted virologic surveillance using mosquitoes and pig serum specimens collected from pig farms in multiple counties in Taiwan during the 2009 and 2010 transmission seasons. Eight pig farms were selected as sites for virologic surveillance (Figure): 3 in the central counties of Taichung and Changhua; 4 in the southern counties of Yulin, Chiayi, and Tainan; and 1 in the eastern county of Hualien. Mosquito pools and swine serum specimens were collected every other week during the JEV transmission season from March 2009 through October 2010.

The mosquito and swine specimens were subjected to viral RNA detection by using a multiplex reverse transcription PCR (RT-PCR). A QIAamp Viral RNA kit (QIAGEN, Hilden, Germany) was used to extract viral RNA from pooled ground mosquitoes or from plasma specimens following the manufacturer’s protocol. Three primer pairs were used in RT-PCR to differentiate GI and GIII JEV (Table). Amplifying and sequence primers were used as was done in our previous study (*10*) to obtain E gene region and the full genomic sequence. The DNA fragment of the correct size was excised, purified with a Viogene gel extraction kit (VIOGENE, Sunnyvale, CA, USA), and sequenced directly by using a Prism automated DNA sequencing kit (Applied Biosystems, Foster City, CA, USA).

**Table:** The 3 primer pairs used in reverse transcription PCR to differentiate genotypes I and III Japanese encephalitis virus, Taiwan, 2009–2010*

Specificity/ name	Sequence, 5′ → 3′	Location†
All JEV		
JE1F	TGTGTGAACTTCTTGGCTTAGTAT	12–35
JE1R	CARCATCTGTTYTCWCCTTTTGA	559–581
GI JEV only		
GIJEVF	CAGTCGCGAGTTTAAACGAC	2000–2019
GIJEVR	CATTCAGTTCGTCCCGCACA	2688–2707
GIII JEV only		
GIIIJEVF	GGATGCTTGGCAGTAACAAC	904–923
GIIIJEVR	AAGTCCACATCCGTTGCC	1281–1298

*JEV, Japanese encephalitis virus; GI, genotype I; GIII, genotype III. †According to JEV strain T1P1 complete genome (GenBank accession no. AF254453).

A total of 62,266 mosquitoes were collected at multiple sites in Taiwan from March 2009 through October 2010. The most common species was *Cx. tritaeniorhynchus* (n = 59,386). Of the 787 mosquito pools collected, 37 pools were JEV-positive by multiplex RT-PCR, and all positive pools contained *Cx. tritaeniorhynchus* mosquitoes. The JEV-positive mosquitoes were collected from central (Taichung and Changhua Counties), southern (Yulin, Chiayi, and Tainan Counties), and eastern (Hualin County) Taiwan (Figure). Unfortunately, JEV was not detected from pig serum specimens, but seroconversion, defined by plaque-reduction neutralizing assay at the 50% reduction titer of >10, was evident among these samples after JEV detection in mosquitoes (data not shown).

**Figure A1:**
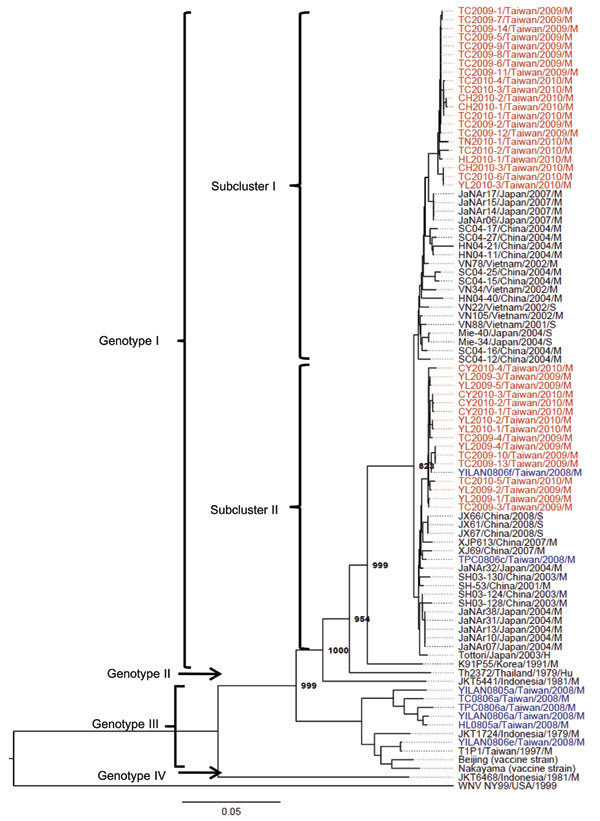
Phylogenetic analysis of Japanese encephalitis virus (JEV) isolates from Taiwan, 2009–2010. The phylogenetic tree construction was based on full-length envelope protein sequences by using the neighbor-joining method. JEV isolates obtained in this study are indicated in red, and isolates obtained by the Taiwan Centers for Disease Control are indicated in blue (*9*). Bootstrap values >700 are shown (1,000 replicates). Scale bar represents nucleotide substitutions per site.

Full-length JEV sequences, including TC2009–1, TC2009–1–3, and YL2009–4 (GenBank accession nos. JF499788–JF499790), were obtained from 3 positive mosquito pools, and a neighbor-joining phylogenetic tree confirmed all 3 viruses belong to GI (data not shown). Additionally, the E gene was sequenced from 37 JEV-positive mosquito pools (GenBank accession nos. JF499791–JF499827). The full-length E gene sequence of these isolates was more closely related to the GI K94P05 strain than to the GIII Nakayama strain; sequence identities ranged from 97.6% to 98.6%, compared with 87.4% to 88.1%, respectively. The E gene tree supports that all 37 isolates detected in this study belong to GI (Figure A1). However, of JEV detected by CDC-Taiwan in 2008, only TPC0806c and YILAN0806f were classified as GI; all others belonged to GIII (*9*).

GI JEV can be classified into various subclusters based on phylogeny (*8*). In this study, 21 of the 37 viruses could be classified as subcluster I and the other 16 as subcluster II. The TPC0806c and YILAN0806f strains, reported by CDC-Taiwan, belong to subcluster II (Figure A1) (*9*). This result indicates that the GI JEV introduced into Taiwan originated from multiple sources.

To understand the origin of the GI JEV introduced into Taiwan, we selected the K91P55 strain as the root virus and constructed a minimum-spanning tree by using BioNumerics software version 5.00 (Applied Maths; Austin, TX, USA). Among the subcluster I JEV isolated in Taiwan, the most pronounced genetic linkages appeared between viruses isolated in Taichung in 2009 and in Japan in 2007. The Taichung isolates (TC2009-2, -5, -6, -8, -9, and -11) were most closely linked to a group of viruses (JaNAr06, 14, 15, and 17) isolated from mosquitoes collected in Nagasaki, Japan, in 2007. Among subcluster II JEV in Taiwan, the TC2009-3 Taichung isolate and the TPC0806c Taipei City isolate were most closely related to JaNAr32-04, which was isolated from mosquitoes collected in Nagasaki, Japan, in 2004. In summary, the JEV GI isolates from Taiwan, most closely related to GI viruses isolated from Nagasaki, Japan, were introduced at least twice into central and once into northern Taiwan.

## Conclusions

GI JEV first appeared in Taiwan in 2008 when GIII viruses were still the dominant circulating genotype in the region. We conclude, on the basis of molecular evidence, that the dominant JEV genotype in Taiwan has switched from III to I. Also, our study suggests that 1) the genotype replacement may have been accomplished within 1 year; 2) the JEV ecology remains unchanged, as evidenced by the involvement of *Cx. tritaeniorhynchus* mosquitoes and of swine in maintaining and circulating GI virus; 3) the introduction of GI JEV strains occurred multiple times, resulting in the detection of subcluster I and II viruses; and 4) the GI JEVs isolated in Taiwan were most closely related to GI viruses isolated from Nagasaki, Japan. Current human and swine vaccines are derived from GIII viruses. Thus, the systemic evaluation of the cross-neutralizing activity of vaccinated human, as well as swine, serum specimens should be used to estimate the protective efficacy of GIII-based vaccine.

## Figures and Tables

**Figure:**
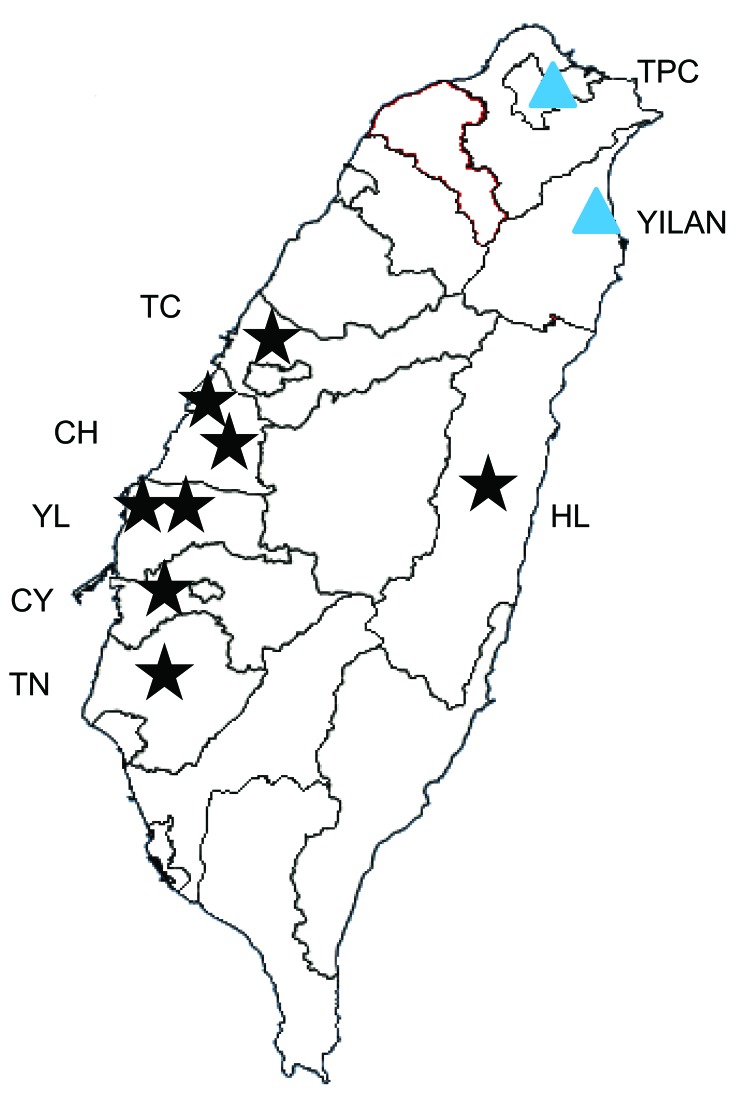
Location of pig farms from which mosquitoes were collected for phylogenetic analysis of Japanese encephalitis virus (JEV) isolates, Taiwan, 2009–2010. Black stars indicate location of pig farms. Blue triangles indicate locations at which genotype I JEV was first detected by the Taiwan Centers for Disease Control (*9*). CH, Changhua County; CY, Chiayi County; HL, Hualien County; TC, Taichung County; TN, Tainan County; TPC, Taipei City; YL, Yulin County.
